# Gut Microbiota‐Derived Bacterial Extracellular Vesicles in COVID‐19: Their Signature and Immunological Impact

**DOI:** 10.1002/jev2.70341

**Published:** 2026-07-23

**Authors:** Aya Ishizaka, Michiko Koga, Tomoya Hayashi, Ken J Ishii, Hiroyuki Yamamoto, Hiroshi Yotsuyanagi, Taketoshi Mizutani

**Affiliations:** ^1^ Division of Infectious Diseases, Advanced Clinical Research Center, The Institute of Medical Science The University of Tokyo Tokyo Japan; ^2^ AIDS Research Center, National Institute of Infectious Diseases Japan Institute for Health Security Tokyo Japan; ^3^ Department of Infectious Diseases and Applied Immunology, IMSUT Hospital of the Institute of Medical Science The University of Tokyo Tokyo Japan; ^4^ The University of Tokyo Pandemic Preparedness, Infection and Advanced Research Center The University of Tokyo Tokyo Japan; ^5^ Division of Vaccine Science, Department of Microbiology and Immunology, The Institute of Medical Science The University of Tokyo Tokyo Japan; ^6^ International Vaccine Design Center, The Institute of Medical Science The University of Tokyo Tokyo Japan; ^7^ Immunology Frontier Research Center (IFReC) Osaka University Osaka Japan; ^8^ Japan Institute for Health Security Tokyo Japan; ^9^ Department of Diagnostic Testing and Technology Research, National Institute of Infectious Diseases Japan Institute for Health Security Tokyo Japan

**Keywords:** bacteria‐derived extracellular vesicles, coronavirus disease 2019, gut microbiota, immune response

## Abstract

Gut microbial dysbiosis has been observed in several diseases. Although causal links and direct effects on host cells remain unclear, bacteria‐derived extracellular vesicles (BEVs) from the gut microbiota may regulate the host immune response. We examined the impact of severe acute respiratory syndrome coronavirus 2 (SARS‐CoV‐2) infection on the gut microbiome and BEVs release, and the effects of released BEVs on cytokine responses in monocyte‐derived cell lines. Fecal samples from 17 patients with coronavirus disease 2019 (COVID‐19) and 20 healthy individuals were collected to isolate bacterial and BEV fractions. Parental BEV‐releasing bacteria were identified from vesicle‐encapsulated bacterial DNA by 16S rRNA gene sequencing. Patients with COVID‐19 exhibited altered gut microbiota composition and the profile of bacterial DNA‐containing BEVs (dcBEVs) release compared to healthy controls. BEVs from patients, but not from healthy individuals, significantly changed cytokine levels in U937 monocyte cells. Following COVID‐19 recovery, dcBEV profiles diverged into two distinct groups: those that retained the capacity to induce cytokines in monocytes and those that lost this functionality. BEVs from single bacterial cultures within families altered after COVID‐19 onset affected the expression of genes in monocytes, primarily immune‐response genes, notably chemokine ligands and G protein‐coupled receptors. SARS‐CoV‐2–induced dysbiosis alters the profile of dcBEVs release, thereby modulating the host immune response and potentially contributing to COVID‐19 pathogenesis.

## Graphical abstract

This study demonstrated that the secretion of bacterial extracellular vesicles (BEVs) changes during COVID‐19‐induced gut‐bacterial dysbiosis, and these alterations in BEV profiles influence the immune cell cytokine response, persisting even after clinical remission. BEVs' effect on immune cells depends on bacterial species, suggesting specific bacterial imbalances may impact disease progression.

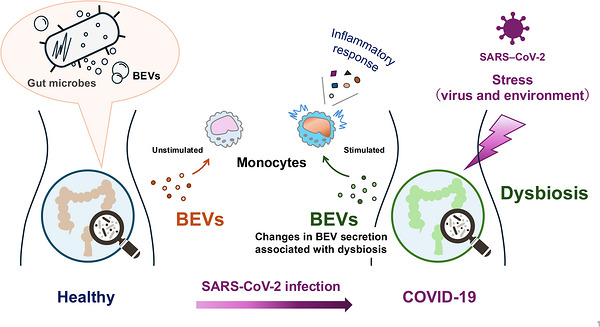

## 1 Introduction

Coronavirus disease 2019 (COVID‐19) is a severe acute respiratory syndrome caused by coronavirus 2 (SARS‐CoV‐2), with most cases resulting in mild illness; nonetheless, severe cases can lead to multi‐organ disease and are potentially fatal. Even after the pandemic, viral infection cases continue to occur, and understanding the factors that influence disease outcomes is crucial from a preventive perspective (Ellwanger et al. 2021). Notably, previous reports suggested that a gut microbiota (dysbiosis) imbalance contributes to COVID‐19 pathogenesis (Lau et al. 2025, Yamamoto et al. 2021).

The gut microbiota primarily resides in the human digestive tract, creating a symbiotic relationship with the host, playing crucial roles in nutrient metabolism and immunomodulation (Jandhyala et al. 2015). Dysbiosis is associated with several infectious diseases (Ishizaka et al. 2025), including SARS‐CoV‐2 infection, and numerous cases of dysbiosis with bacterial diversity alterations have been reported in COVID‐19 pathogenesis (Lau et al. 2025). Because bacteriophages, viruses that infect and replicate within bacteria, are also linked to this gut microbiota alteration, both the bacteria and the overall dynamics of intestinal microorganisms may be altered with COVID‐19 onset (Ishizaka et al. 2025, Lu et al. 2021). Importantly, these alterations may relate to COVID‐19 pathogenesis via bidirectional crosstalk between gut and lung tissues, commonly known as the gut‐lung axis (de Oliveira et al. 2021). The primary bacterial alteration after disease onset is reduced short‐chain fatty acid–producing bacteria, which have been linked to increased intestinal epithelial vulnerability and disturbed intestinal immune homeostasis (Ishizaka et al. 2024, Zhang et al. 2023). This change is common to many intestinal diseases (Zhang et al. 2023). Furthermore, our observations indicate prolonged intestinal microbiota abnormalities, potentially linked to post‐acute sequelae of SARS‐CoV‐2 (PACS) (Ishizaka et al. 2024). Numerous studies on COVID‐19 pathology support the notion that such changes in symbiotic microorganisms contribute to PACS (Lau et al. 2025).

Despite the accumulation of these observational perspectives, the causal relationship between gut dysbiosis and host immune response in COVID‐19 remains unclear. Recently, there has been growing interest in the immunological function of bacterial extracellular vesicles (BEVs), which range in size from 20 to 400 nm and are released by intestinal bacteria (Nie et al. 2024). Notably, BEVs contain bacteria‐derived nucleic acids and proteins, and their physiological functions in bacterial proliferation, metabolism, and signal transduction are subjects of active research (Ishizaka et al. 2021). Moreover, their potential impact on human health, particularly through interactions that affect immunological gene expression, is becoming increasingly evident (Dominguez Rubio et al. 2022). Previous reports suggest two primary categories of BEVs: those that contain nucleic acids and those that do not or contain only trace amounts (Toyofuku et al. 2023). BEVs lacking nucleic acids are generated when live Gram‐negative bacteria undergo a process known as blebbing, in which a portion of their outer membrane is pinched off. These vesicles predominantly comprise outer membrane proteins and lipids owing to the difficulty in incorporating cytoplasmic contents during this process. Conversely, bacterial DNA‐containing BEVs (dcBEVs) are formed when bacteria die or when the cell wall is disrupted, resulting in the encapsulation of cytoplasmic DNA and RNA within the membrane. Consequently, while dcBEVs represent a specific, relatively small subset of the total released BEV population, their encapsulated genetic material provides a unique and highly informative analytical “window” into the active functional dynamics of the gut microbiota. Although this approach has an inherent limitation in that it does not capture non‐DNA‐containing vesicles, it offers a distinct advantage by allowing the targeted high‐throughput sequencing of vesicle‐associated bacterial DNA, thereby directly linking specific microbial taxa to the circulating vesicle pool under pathological conditions.

We have previously reported that dysbiosis occurs after COVID‐19 onset, with specific microbiota changes correlated with blood cytokine levels (Mizutani et al. 2022). We hypothesized that changes in BEVs release and quality associated with microbial imbalance in COVID‐19 may affect the host immune response and homeostasis. To evaluate this hypothesis, this study focused on the 16S rRNA gene contained in a portion of BEV particles and investigated the correlation between gut bacterial changes during COVID‐19 onset and dcBEVs release. This study aimed to clarify the characteristics of dcBEVs release following COVID‐19 onset and to elucidate its immunological functions.

## 2 Materials and Methods

### 2.1 Subject Recruitment and Sample Collection

In 2020, fecal samples were collected from 17 COVID‐19 patients at the Institute of Medical Science, The University of Tokyo Hospital (Table S1). The patients were divided into three groups based on their symptoms in our previous study (Mizutani et al. 2022). Patients with symptoms such as fever, cough, and sore throat, but no CT scan signs of breathing trouble or pneumonia, were considered mild cases. Those with CT scan signs of pneumonia and symptoms such as fever and breathing trouble, but with oxygen levels of 94% or higher, were called moderate cases. Patients with CT signs of pneumonia and oxygen levels < 94% were considered severe cases (Table S1). Clinical information on patients is shown in Table S2. The patient had a regular diet during their hospitalization. The samples were collected during the early stages of the COVID‐19 pandemic when no effective vaccine against SARS‐CoV‐2 existed. Patients were hospitalized and treated according to the regulations of the time, and they provided stool samples during their hospitalization (Mizutani et al. 2022). The fecal samples were collected within three weeks of the onset of COVID‐19. Some patients also provided stool samples after discharge. For this analysis, 20 healthy adults were randomly selected from a pre‐pandemic cohort of 50 individuals recruited in 2018. Subjects who had used antibiotics within the two weeks prior to sampling were excluded. The samples were stored at ‐80°C until DNA was extracted.

### 2.2 Protocol for Extracting Bacterial Fraction From Fecal Specimens

Isolation of the bacterial fraction from stool samples was performed using a previously established method (Mizutani et al. 2022). Specifically, 1 g of a frozen stool sample was thawed and thoroughly mixed with SM‐plus Buffer, which contained 100 mM NaCl, 50 mM Tris‐HCl (pH 7.4), 8 mM MgSO_4_, 5 mM CaCl_2_, and 0.01% gelatin. The mixture was then centrifuged, and the supernatant was discarded. The remaining solid material was combined with SM‐plus buffer, stirred, and passed through a 100 µm cell strainer (Corning, Inc., Corning, NY, USA). The filtered sample was used as the bacterial fraction.

### 2.3 Microbial Culture

*Bifidobacterium longum* (*B. longum*) and *Bacteroides fragilis* (*B. fragilis*) were cultured anaerobically at 37°C using the AnaeroPack Kenki system (Mitsubishi Gas Chemical Co., Inc., Tokyo, Japan) and incubated overnight. The culture medium for *B. longum* was BD Difco Lactobacilli MRS Broth (BD Biosciences, NJ, USA), whereas *B. fragilis* was cultured in BD BBL Brain Heart Infusion (BHI) broth (BD Biosciences) supplemented with 5 µg/ml hemin (FUJIFILM Wako Life Science, Osaka, Japan).

### 2.4 Isolation of Bacterial Extracellular Vesicles

As a preprocessing step, stool samples were homogenized with PBS at a 1:10 weight/volume ratio and centrifuged at 6000× g for 15 min to remove debris. For bacterial cultures, the bacterial cells were removed by centrifugation at 6000 × g for 15 minutes. Each supernatant was subsequently filtered through a 0.22 µm filter (Merck Millipore, Billerica, MA, USA). For further purification of BEVs, the filtrates were centrifuged at 100,000 × g for 3 hours using an ultracentrifuge (AVANTI JXN‐30, JS‐24.15 rotor; Beckman Coulter, Brea, CA, USA). The resulting precipitate was resuspended in 5 mL of PBS and then concentrated to 0.5 mL using an Amicon Ultra‐4, PLGC 10 kDa (Merck Millipore). This concentrated solution underwent size‐exclusion chromatography with Sepharose 2B beads (Sigma‐Aldrich, St. Louis, MO, USA). The eluted fractions were collected into 20 fractions of 0.5 mL each. Following SDS‐PAGE, the eluted proteins were stained with Oriole Fluorescent Gel Stain reagent (Bio‐Rad, Hercules, CA, USA). The eluate fractions containing BEVs were collected and concentrated using an Amicon Ultra‐4 PLGC 10 kDa filter (Merck Millipore). The resulting concentrate was then used as BEVs.

### 2.5 Nanoparticle Tracking Analysis for BEVs

The size and count of bacterial extracellular vesicles were determined using a Nanosight NS300 (Quantum Design, San Diego, CA, USA) through nanoparticle tracking analysis. Additionally, the vesicles were observed with an atomic force microscope NanoWizard4 (Bruker Corporation, Billerica, MA, USA).

### 2.6 DNA Extraction, Amplification, and Next‐Generation Gene Sequencing

We extracted DNA from the bacterial fraction derived from the fecal sample, as per a previously described protocol (Ishizaka et al. 2021). Briefly, DNA was extracted from both bacterial and BEV fractions obtained from fecal samples using the phenol‐chloroform method. Subsequently, the hypervariable V3‐V4 region of the 16S rRNA gene was amplified. 16S rRNA gene libraries were prepared following the 16S Metagenomics Sequencing Library Preparation Guide (Illumina, San Diego, CA, USA, part no. 15044223 Rev. B), adhering to the specified procedure. Sequencing was conducted on an Illumina MiSeq (Illumina) using the MiSeq Reagent Kit v3 (600 cycles) spiked with 20% PhiX (Illumina).

### 2.7 Pipeline for 16s rRNA Data Analysis

Bacterial 16S sequences were subjected to quality filtering, denoising, and analysis using Quantitative Insights Into Microbial Ecology 2 (QIIME 2) (Bolyen et al. 2019). Paired‐end reads were processed for amplicon sequence variants using DADA2 (Callahan et al. 2016). The resulting amplicon sequence variants were classified based on the SILVA database (release 132) (Quast et al. 2013). The V3‐V4 region of the 16S rRNA gene was trimmed using the naïve Bayesian classification method (Pedregosa et al. 2011).

### 2.8 Fungal Genomic PCR and SARS‐CoV‐2 RNA Detection

Fungal genomic PCR was performed by amplifying the internal transcribed spacer (ITS) region 1 of the fungi. Total DNA from the BEV fraction was used as template, and PCR was performed according to the ITS1 amplification protocol provided by Illumina (https://support.illumina.com/downloads/fungal‐metagenomic‐sequencing‐demonstrated‐protocol‐1000000064940.html). SARS‐CoV‐2 RNA was detected in BEVs according to the protocol of the Takara SARS‐CoV‐2 Direct PCR Detection Kit (Takara Bio Inc., Shiga, Japan). Specifically, the coding regions of the SARS‐CoV‐2 N protein (N1 and N2) were amplified by quantitative PCR.

### 2.9 Cell Culture and Co‐Culture Experiments of BEVs and Cultured Cells

The human monocytic cell line U937 was obtained from the JCRB Cell Bank (National Institutes of Biomedical Innovation, Health and Nutrition, Osaka, Japan). For *ex vivo* validation, human peripheral blood mononuclear cells (PBMCs) were isolated from whole blood samples obtained from healthy volunteers using density gradient centrifugation with SepMate (STEMCELL Technologies, Vancouver, BC, Canada). Both U937 cells and isolated PBMCs were cultured in RPMI‐1640 medium (Wako Pure Chemical Industries, Osaka, Japan) supplemented with either 10% fetal calf serum (for U937) or 10% autologous serum (for PBMCs), at 37°C in a humidified atmosphere containing 5% CO_2_.For stimulation experiments, both cell types were seeded at a density of 50,000 cells and treated with BEVs at the indicated concentrations for 24 h. Following incubation, the culture supernatants were harvested for subsequent cytokine assays. For U937 cells, the stimulated cells were additionally collected and subjected to total RNA extraction for subsequent RNA sequencing analysis.

### 2.10 Flow Cytometric Analysis of BEVs

For the preparation of human‐derived EVs, 293F cells (ATCC, Manassas, VA, USA) were cultured in serum‐free FreeStyle 293 Expression Medium (Gibco, Grand Island, NY, USA) at a density of 3 × 10^5^ cells/mL in a 10 cm dish for 72 h at 37°C under 5% CO_2_. The conditioned medium was first centrifuged at 400 × g for 5 min at 4°C to remove cells. The supernatant was then subjected to ultracentrifugation at 120,000 × g for 1 h at 4°C to pellet EVs. The resulting pellet was resuspended in PBS(‐) and analyzed by flow cytometry. Single‐particle analysis was performed on the CytoFLEX LX Flow Cytometer (Beckman Coulter, CA, USA) with a VSSC gain of 90 and VSSC‐H threshold of 360, which yielded approximately 6,000 background events per second. VSSC intensities corresponding to particle sizes of 100, 200 and 500 nm were determined using 100 nm FluoSpheres Carboxylate‐Modified Microspheres (Thermo Fisher, Waltham, MA, USA) and 200 and 500 nm Fluoresbrite YG Carboxylate Microspheres (Polyscience, Warrington, PA, USA). For detection of human‐derived EVs, the BEV suspension was mixed with an equal volume of CellMask deep red plasma membrane stain (Thermo Fisher) to give a working concentration of 1:10,000 of the stock solution. After incubation for 5 min, PE ‐conjugated anti‐CD9, anti‐CD63, anti‐CD81 or isotype control antibody (Biolegend, San Diego, CA, USA) was added at a working concentration of 1 µg/mL, followed by incubation at 4°C for 20 min. For detection of nucleic acids‐containing BEVs, the BEV suspension was incubated with or without Benzonase (Sigma‐Aldrich), DNase I or RNase A (NIPPON GENE, Toyama, Japan) at 1:100 dilution of the respective stock solutions at 37°C for 30 min. Samples were then mixed with CellMask deep red plasma membrane stain as described above, followed by the staining with SYTO 9 (Thermo Fisher, MA, USA) at a final concentration of 5 µM at 4°C for 20 min. Stained BEVs were subsequently diluted 40‐fold in PBS to minimize background fluorescence and measured with CytoFLEX LX at a flow rate of 26 µL/min. Data was analyzed using FlowJo v10 software (BD Biosciences, NJ, USA).

### 2.11 Cytokine and Chemokine Assays

The concentrations of cytokines and inflammatory mediators in the collected culture supernatants from U937 cells and PBMCs were quantified using the Bio‐Plex Pro Human Inflammation 1, 37‐Plex Panel (#171AL001M, Bio‐Rad Laboratories, Hercules, CA, USA) according to the manufacturer's instructions. Fluorescence signals were acquired and analyzed using the Bio‐Plex system (Bio‐Rad Laboratories).

### 2.12 Bacterial Endotoxin Detection in BEV Samples

Bacterial endotoxin levels in BEVs derived from healthy donors (*n* = 14) and patients (*n* = 14) were measured using the ToxinSensor Chromogenic LAL Endotoxin Assay Kit (GenScript, Piscataway, NJ, USA), according to the manufacturer's instructions. Endotoxin concentrations (Endotoxin Unit; EU, reflecting biological activity) were normalized per 2,000 BEV particles.

### 2.13 RNA Sequence and Gene Ontology (GO) Analysis

Total RNA was extracted using the miRNeasy Tissue/Cells Advanced Mini Kit (QIAGEN, Hilden, Germany) according to the manufacturer's instructions. The purity, concentration, and integrity of total RNA were confirmed using a Nanodrop spectrophotometer (Thermo Fisher, MA, USA). RNA sequencing was performed by Gene Nex (http://gene‐nex.com). Specifically, sequencing analysis was conducted using the Novaseq X Plus platform, generating 150‐bp paired‐end reads. Raw sequencing reads were first pre‐processed for quality control and adapter trimming using fastp. Transcript abundances were estimated using Salmon against [human transcriptome (GCF_000001405.40_GRCh38.p14)]. Gene abundances were then calculated from transcript abundances using the tximport package. Differential expression analysis was performed using the edgeR package after the filterByExpr function was applied to remove low‐expressed genes. The genes with |log2 Fold Change |  > 1 and false discovery rate (FDR) < 0.05 were considered differentially expressed. Functional enrichment analysis was performed on differentially expressed genes (DEGs) using the clusterProfiler package against GO (https://geneontology.org/) and WikiPathways (https://www.wikipathways.org/). GO terms and pathways with an adjusted *p*‐value < 0.05 were considered statistically enriched. This analysis was performed on OlvTools (https://olvtools.com/, BxINFO, Tokyo, Japan)

### 2.14 Quantitative Real‐Time PCR (qPCR)

qPCR was performed using a CFX96 Real‐Time PCR Detection System (Bio‐Rad Laboratories) with the KAPA SYBR FAST qPCR Kit (KAPA Biosystems, Wilmington, MA, USA). All reactions were performed in triplicate, and threshold cycle (Ct) values were determined using the system's software. The mRNA expression levels of CXCL10, CXCL8, and GAPDH (as an internal control) were quantified using the following specific primer pairs: CXCL10 forward, 5'‐GGTGAGAAGAGATGTCTGAATCC‐3' and reverse, 5'‐TGCAGTGCTTCCAAGGATGGAC‐3'; CXCL8 forward, 5'‐CTCTCTTGGCAGCCTTCCT‐3' and reverse, 5'‐GGGTGGAAAGGTTTGGAGTA‐3'; and GAPDH forward, 5'‐CTCTGCTCCTCCTGTTCGAC‐3' and reverse, 5'‐TTAAAAGCAGCCCTGGTGAC‐3'.

### 2.15 Statistical Analysis

Beta diversity patterns were visualized via Principal Coordinate Analysis (PCoA) based on Weighted UniFrac distances using the QIIME 2 platform. Other statistical analyses were performed using GraphPad Prism 10 (GraphPad Software, Boston, MA, USA). Multiple Mann–Whitney U tests were applied, and the Benjamini–Hochberg procedure was used to control the FDR; adjusted *p*‐values < 0.05 were considered statistically significant.

## 3 Results

### 3.1 BEVs Purification From Fecal Samples

**FIGURE 1:**
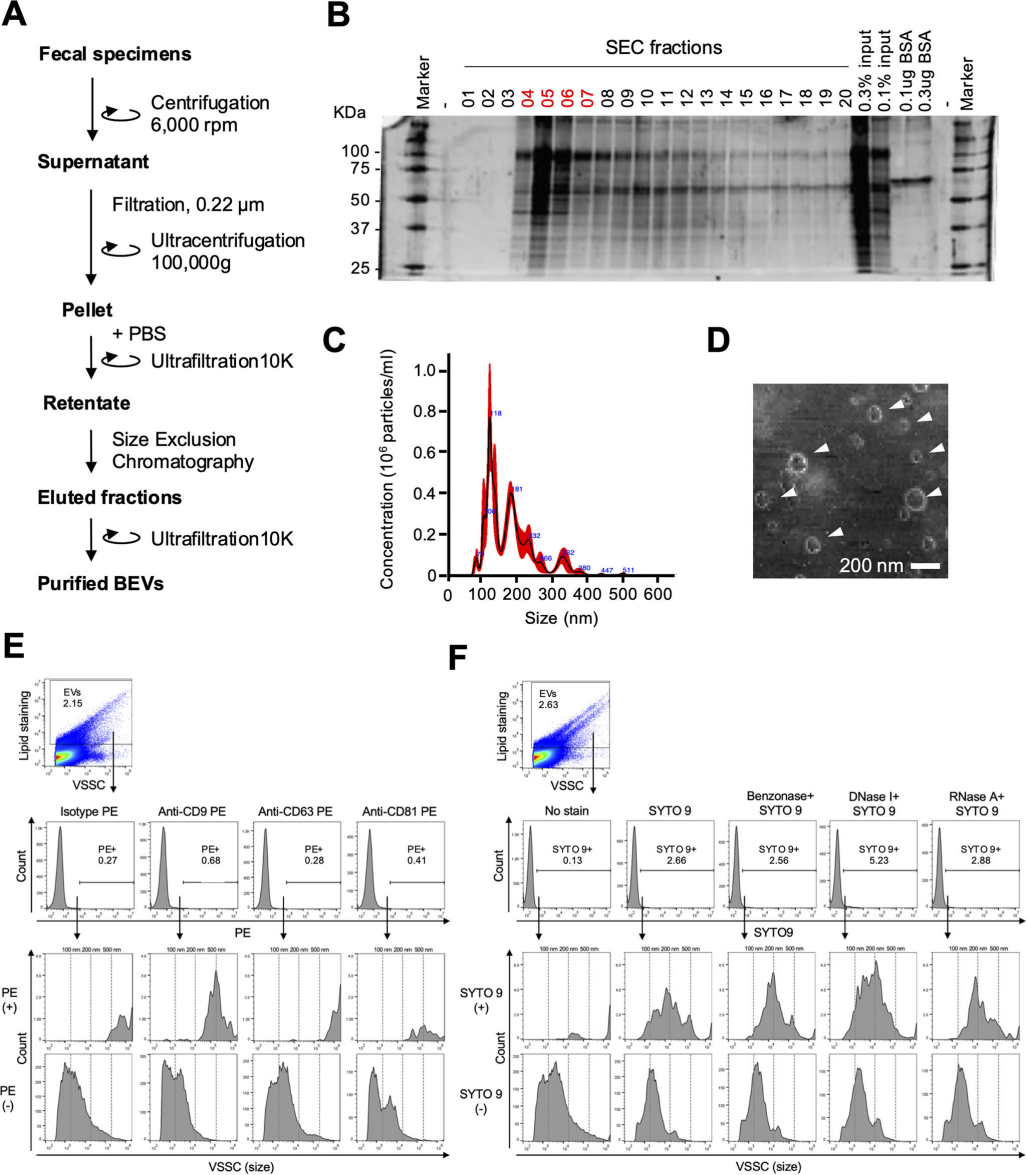
**Purification and biophysical characterization of bacterial extracellular vesicles (BEVs) from human fecal samples**. (A) Schematic of the BEV purification workflow. (B) SDS‐PAGE of fractions (No. 1–20) collected via size‐exclusion chromatography (SEC). Red box/labels indicate BEV‐enriched fractions (No. 4–7). BSA: Bovine Serum Albumin. (C) Particle size distribution of post‐SEC BEVs measured by nanoparticle tracking analysis (NTA). The red line and shaded area represent the mean and SEM (N=3). (D) Atomic force microscopy (AFM) of purified BEVs (scale bar = 200 nm). White arrowheads denote representative vesicles. (E, F) Nanoflow cytometric analysis of (E) human‐derived EVs and (F) nucleic acid‐containing vesicles within the BEV‐enriched fraction. Histogram plots show the particle size (nm) distribution.

We hypothesized that SARS‐CoV‐2 infection‐induced dysbiosis alters the BEVs. We compared the BEV profiles of fecal samples collected from 17 patients with COVID‐19, and 20 healthy controls (HC) enrolled in this study. Figure 1A displays a schematic diagram of the method used to purify BEVs from the fecal samples. The purification scheme involved filtering the supernatant of the fecal sample and then combining ultracentrifugation and size‐exclusion chromatography to isolate BEVs from the fecal samples. A photograph of the gel obtained using size‐exclusion chromatography is provided in Figure 1B. Purified BEVs were analyzed using a nanosized microscope, which demonstrated multiple peaks ranging from approximately 80 to 400 nm (Figure 1C). The full‐size distribution profiles and mean diameters of the BEVs for the samples are provided in Figure S1. Atomic force microscopy observations confirmed the presence of numerous heterogeneous particles (Figure 1D), and the data indicated that BEVs derived from feces were likely heterogeneous in size, originating from multiple parental bacteria residing in the intestinal tract. Next, we assessed the contamination rate of human‐derived EVs within the BEV fractions using flow cytometry. To assess the specificity and sensitivity of our detection method, we first performed validation experiments using human‐derived EVs isolated from two distinct cell lines as positive controls: COLO201 (human colon adenocarcinoma) and 293F (human embryonic kidney). Although the expression levels of the EV markers (CD9, CD63, and CD81) varied between these cell lines, the detection system consistently identified these markers in the positive control samples (Figure S2), confirming sufficient assay sensitivity. We then applied this method to the BEV fractions, where particles expressing CD9, CD63, or CD81 accounted for less than 1% of the total detected particles (Figure 1E), indicating that contamination by human‐derived EVs in our BEV preparations is minimal. This observation is consistent with previous reports indicating a low rate of human‐derived EV contamination in fecal samples (Takano et al. 2025). In addition, fungal EVs and SARS‐CoV‐2 viral particles, which may be present as nanoparticles, were undetected in any of the participants' BEV samples using PCR analysis. When the obtained nanoparticles were stained with SYTO 9 (a nucleic acid stain) to assess the presence of nucleotides in BEVs, approximately 5 % of the lipid‐stained nanoparticles were positive for SYTO 9 (Figure 1F). This observation is in line with the previous report that found it difficult to detect nucleic acid encapsulation using fluorescent staining (Takano et al. 2025). Pre‐treatment of BEVs with nucleases did not affect the proportion of SYTO 9^+^ particles, suggesting most nucleic acids were encapsulated within the vesicles and were therefore protected from enzymatic degradation (Figure 1F). The SYTO 9^+^ fraction exhibited a broad size distribution ranging from 100 to 500 nm, while the SYTO 9^−^ fraction showed a relatively small size centered around 100 nm (Figure 1F) (Toyofuku et al. 2023).

### 3.2 Bacterial DNA Containing‐BEV Release Levels Change With COVID‐19

**FIGURE 2:**
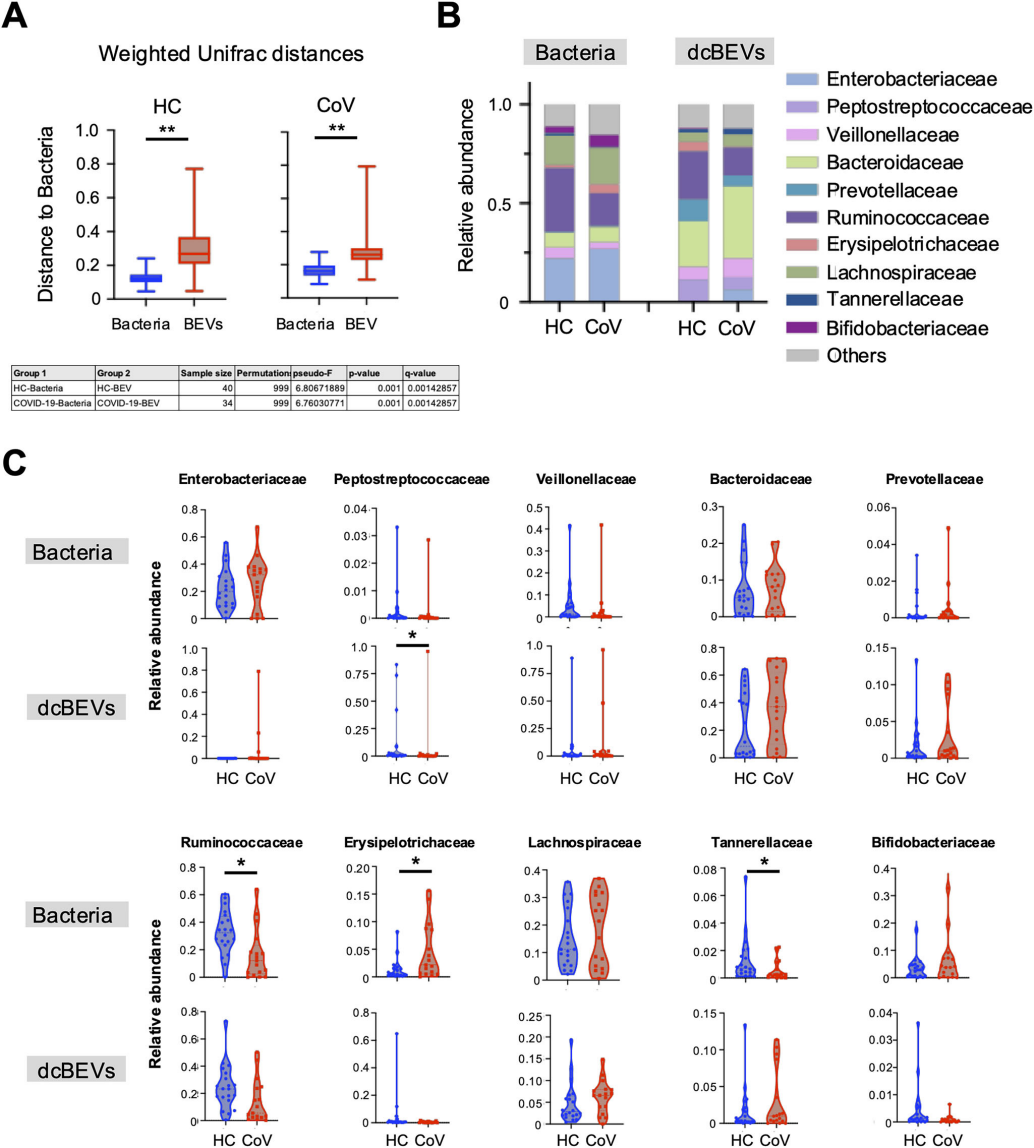
**Impact of COVID‐19 on gut microbiota composition and dcBEV dynamics**. (A) Principal coordinate analysis (PCoA) of weighted UniFrac distances comparing the beta diversity of parental bacteria and their released dcBEVs in fecal samples from healthy controls (left) and COVID‐19 patients (right). (B) Relative abundance of the top 10 parental bacteria and their corresponding dcBEVs at the family level. (C) Comparison of the relative abundance of gut bacteria (top) and their released dcBEVs (bottom) between healthy controls and COVID‐19 patients. Data are presented as violin plots showing the median (central horizontal bar) and interquartile range (IQR). ^*^ *p* < 0.05, ^**^ *p* < 0.01 (Mann–Whitney U test).

As certain BEV particles harbor parent‐bacterial nucleotides (Figure 1F), 16S rRNA genes can be amplified and identified via next‐generation sequencing. Consequently, we performed dcBEV‐based sequence analysis as a proxy for monitoring release changes during COVID‐19 progression. We first evaluated the diversity of the gut microbiota and released dcBEVs in both HC and patients with COVID‐19. In the HC group, beta diversity analysis revealed significant differences between the gut microbiota and the dcBEVs‐releasing microbiota (Figure 2A, left); similar results were observed in the COVID‐19 cohort (Figure 2A, right). These results suggest that gut microbiota composition does not directly reflect the abundance of released BEVs, likely due to taxon‐specific variations in BEV production. To further investigate this, Figure 2B displays the abundance of the top 10 dcBEVs detected in stool and the abundance of their parent microbiota (family level) in both HC and COVID‐19; statistical values are presented in Table 1. Figure 2C compares the individual relative abundances of gut bacterial families and their corresponding dcBEVs between HCs and COVID‐19 patients. Notably, the abundance of these taxa (e.g., Ruminococcaceae, Erysipelotrichaceae, Tannerellaceae, and Peptostreptococcaceae) did not consistently correlate with their respective dcBEV release levels. This indicates that the efficiency of dcBEV production differs among bacterial taxa; such a discrepancy implies that dcBEV release is regulated by mechanisms independent of bacterial biomass.

**TABLE 1:** Prevalance and an abundance of top 10 dcBEVs and their secreting parent microbiota detected in fecal samples.

	Microbiota						dcBEVs					
	Prevalence (%)		median (IQR)		*p* value	adj. *p *value	Prevalence (%)		median (IQR)		*p* value	adj. *p *value
	HC	COVID	HC	COVID			HC	COVID	HC	COVID		
**Enterobacteriaceae**	100	94.1	0.222 (0.097–0.319)	0.323 (0.199–0.379)	0.4065	0.546016	85.0	88.2	0.0002 (0.0001–0.0003)	0.0005 (0.0001–0.0041)	0.1652	0.2129
**Peptostreptococcaceae**	70.0	41.2	0.0004 (0.0000–0.0028)	0.0001 (0.0000–0.0006)	0.1144	0.222619	100	65.0	0.0051 (0.0022–0.0267)	0.0034 (0.0000–0.0129)	0.0056	0.0107*
**Veillonellaceae**	90.0	94.1	0.0408 (0.0053–0.0835)	0.0027 (0.0007–0.0199)	0.1325	0.222619	95.0	88.2	0.0073 (0.0004–0.0267)	0.0128 (0.0002–0.0349)	0.9703	0.9110
**Bacteroidaceae**	100	100	0.0466 (0.0135–0.0772)	0.0822 (0.0223–0.1160)	0.7066	0.593535	100	100	0.1141 (0.0289–0.4122)	0.4341 (0.2854–0.6578)	0.1174	0.2089
**Prevotellaceae**	65.0	52.9	0.0004 (0.0000–0.0044)	0.0015 (0.0000–0.0049)	0.704	0.593535	85.0	88.2	0.0061 (0.0002–0.1337)	0.0256 (0.0011–0.1412)	0.5708	0.5997
**Ruminococcaceae**	100	100	0.3442 (0.2612–0.4519)	0.0903 (0.0152–0.1833)	0.0073	0.0305*	100	100	0.2332 (0.1738–0.3449)	0.1022 (0.0323–0.2373)	0.0259	0.0611
**Erysipelotrichaceae**	100	100	0.0067 (0.0020–0.0154)	0.0301 (0.0071–0.0864)	0.0044	0.0305*	95.0	94.1	0.0052 (0.0019–0.0134)	0.0011 (0.0002–0.0061)	0.0226	0.0611
**Lachnospiraceae**	100	100	0.1127 (0.0545–0.2621)	0.1535 (0.0514–0.2751)	0.6191	0.593535	100	100	0.0287 (0.0191–0.0577)	0.0701 (0.0401–0.0914)	0.1267	0.2089
**Tannerellaceae**	100	94.1	0.0072 (0.0035–0.0197)	0.0025 (0.0005–0.0125)	0.0169	0.0472*	100	88.2	0.0036 (0.0016–0.0181)	0.0127 (0.0031–0.0941)	0.4644	0.5593
**Bifidobacteriaceae**	100	88.2	0.0355 (0.0066–0.0485)	0.0375 (0.0031–0.0713)	0.4924	0.546016	90.0	64.7	0.0013 (0.0005–0.0055)	0.0010 (0.0001–0.0032)	0.0399	0.0611

*p < 0.05

### 3.3 Cytokine Secretion Induction in Monocyte‐Derived Cell Lines by BEVs Derived From Patients With COVID‐19

**FIGURE 3:**
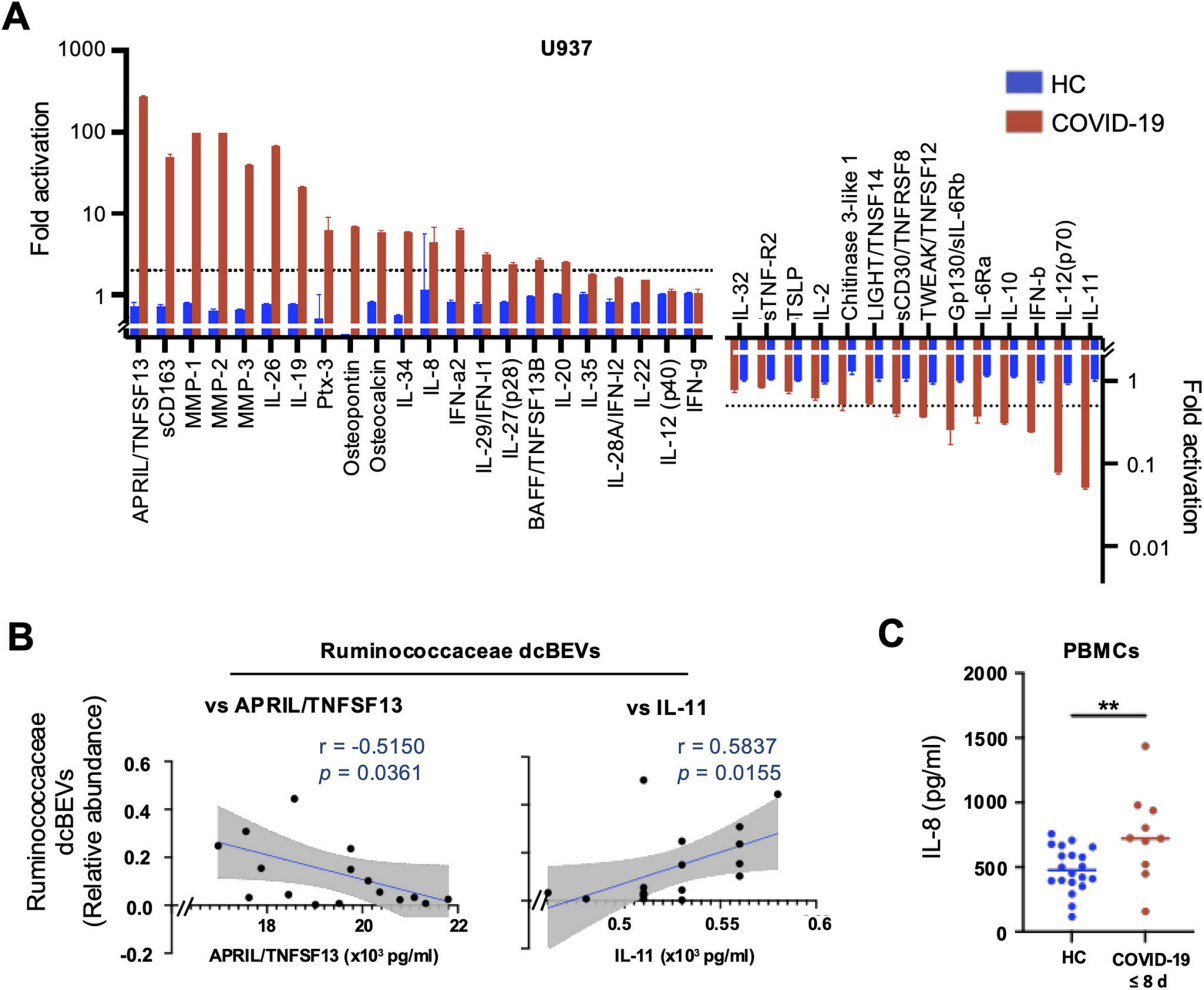
**Altered cytokine responses in U937 cells induced by COVID‐19 patient‐derived BEVs**. (A) Fold‐change in cytokine levels in U937 cell culture supernatants after 24‐hour stimulation with BEVs (2,000 particles/cell) from healthy controls (HC) and COVID‐19 patients, relative to untreated controls. Data are shown as median with 95% confidence interval (CI). The dashed line indicates a 2‐fold change threshold. (B) Correlation between cytokine levels (APRIL/TNFSF13 and IL‐11) secreted from U937 cells following COVID‐19‐derived BEV stimulation and the relative abundance of *Ruminococcaceae* dcBEVs in the corresponding fecal samples. (C) IL‐8 levels in primary PBMC culture supernatants with BEVs from HC and COVID‐19 patients (**p < 0.01).

Given the observed alterations in fecal dcBEV dynamics following COVID‐19 onset, we examined the functional effects of these BEVs on host immune cells. To model the early, intravascular encounters between circulating blood monocytes and systemic BEVs before terminal tissue differentiation, we utilized the human monocytic cell line U937 in its undifferentiated state as an initial screening platform. The cells were treated for 24 h with purified BEVs from either HC (HC‐BEVs) or COVID‐19 patients (COVID‐19‐BEVs). Subsequently, we analyzed the cell culture medium using a multiplex cytokine enzyme‐linked immunosorbent assay (ELISA) system to examine the inflammatory cytokine responses. Culture medium from untreated U937 cells served as a control (fold activation = 1), and the altered cytokine levels are displayed in Figure 3A. U937 cell treatment with HC‐BEVs resulted in minimal changes, with cytokine levels varying from 0.5‐ to 2‐fold. Conversely, the COVID‐19‐BEVs triggered significant pro‐inflammatory alterations (Figure 3A). A comprehensive list of cytokine expression levels, change ratios, and adjusted *p*‐values is provided in Table S3. While bacterial endotoxins are potential drivers of immunogenicity (Bonhomme et al. 2024), their quantification in each BEV showed no significant differences between healthy donors and patients (Figure S3). Regarding the specific BEVs involved in cytokine fluctuations, Ruminococcaceae dcBEVs correlated with APRIL/TNFSF13 and interleukin (IL)‐11 levels. (Figure 3B). Because Ruminococcaceae dcBEV levels showed a decreasing trend after COVID‐19 onset (Figure 2C), the consistency between these BEVs changes and the observed cytokine changes suggests that specific BEVs may exert physiological effects on immune cells. To further validate the physiological relevance of these findings in a more clinically relevant *ex vivo* model, we examined the effects of these BEVs on human PBMCs. While the overall cytokine responses in PBMCs were more restricted compared to those observed in the U937 screening platform, stimulation of PBMCs with COVID‐19‐BEVs during the acute phase of COVID‐19 led to a significant and selective increase in the secretion of IL‐8 compared to those treated with HC‐BEVs (Figure 3C). These results demonstrate that patient‐derived BEVs directly induce a distinct IL‐8 secretion not only in the monocytic cell line but also in human primary immune cells.

### 3.4 Dynamics of Gut Microbiome and BEVs During COVID‐19 Recovery: Impact on Cytokine Levels

**FIGURE 4:**
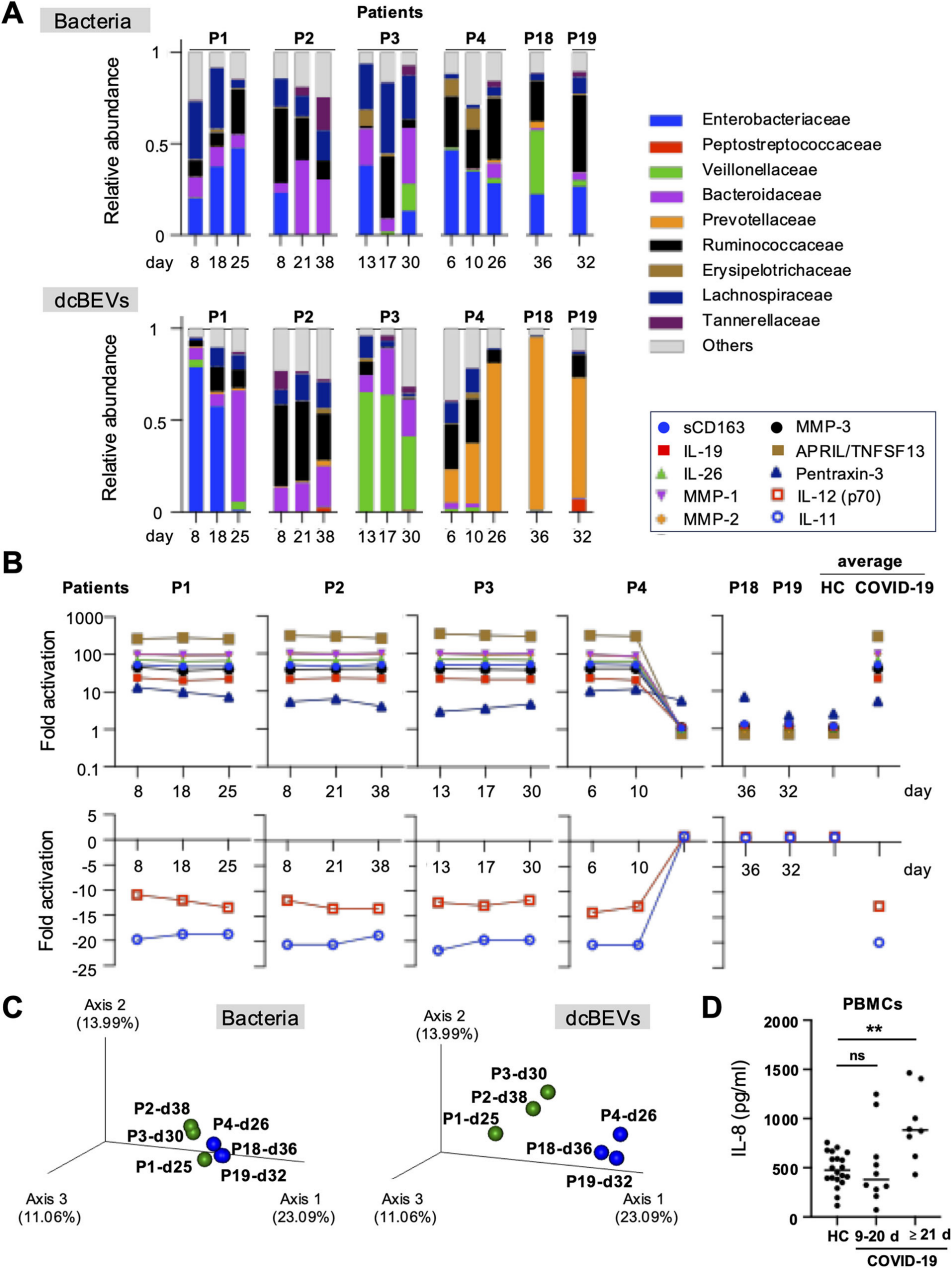
**Comparative analysis of gut bacterial abundance and dcBEV dynamics**. (A) Relative abundance of nine major gut bacterial taxa (upper) and their released dcBEVs (lower) within the same fecal samples. (B) Longitudinal fold‐changes in cytokine levels from U937 cells stimulated with BEVs collected from COVID‐19 patients (*N* = 6) at multiple time points. Cytokine levels are normalized to untreated controls (baseline = 1). The horizontal axis denotes days post‐symptom onset. (C) Three‐dimensional principal coordinate analysis (3D‐PCoA) of weighted UniFrac distances for gut microbiota (left) and released dcBEVs (right) in six patients during the post‐recovery phase (d: day). (D) IL‐8 levels in supernatants of primary human PBMCs stimulated with BEVs from healthy controls (HC) and COVID‐19 patients collected during the intermediate (9–20 days post‐onset) and late (≥ 21 days post‐onset) phases. ns, not significant, **p < 0.01.

Subsequently, we conducted a longitudinal analysis of the alterations in both the gut microbiota and dcBEV profiles of each patient (*n* = 4) who provided multiple stool samples from the onset of COVID‐19. The gut microbiota was predominantly composed of Enterobacteriaceae, Bacteroidaceae, and Ruminococcaceae (patients P1‐P4, Figure 4A top). Nonetheless, dcBEV abundance demonstrated individual variations (Figure 4A bottom). As observed in Figure 3, co‐culturing these BEVs with U937 cells resulted in fluctuations in cytokine levels, which were also observed in samples collected 25 days or more post‐onset (patients P1–P3, Figure 4B and Table S4). Notably, in BEVs derived from patient P4 at 26 days post‐onset, cytokine fluctuation levels were normalized to those of BEVs from healthy individuals. Similarly, in patients P18 and P19, 1 month after onset, BEVs exhibited cytokine secretion induction effects in U937 cells equivalent to those in healthy individuals. These samples contain a high dcBEV proportion derived from Prevotellaceae (Figure 4A bottom). At the genus level, dcBEVs derived from Prevotellaceae detected in patients P4 and P18 were primarily released by *Prevotella 9*, whereas in patient P19, they were primarily released by the *Prevotella* family NK3B31 group (Table S5). Next, principal component analysis of the gut microbiota and released dcBEVs was performed for six post‐recovery COVID‐19 patients (samples collected 25 days or more post‐onset). Between patients P1–P3, who exhibited cytokine fluctuations, and patients P4, P18 and P19, who had cytokine levels similar to those of healthy individuals, the distances between the principal components of the gut microbiota were identical (Figure 4C left). Contrastingly, their dcBEV profiles exhibited distinct separation in the space (Figure 4C right). Based on these observations, individual differences in the quality of BEVs may occur depending on the time elapsed since the onset of COVID‐19 and may have different effects on immune cells. To validate these longitudinal dynamics in primary immune cells, we evaluated IL‐8 secretion from human PBMCs treated with these patient‐derived BEVs over time. While BEVs collected between 9–20 days post‐onset generally induced IL‐8 levels comparable to those treated with HC‐BEVs, a subset of specimens retained the capacity to induce substantial IL‐8 secretion (Figure 4D). Notably, this persistent IL‐8‐inducing capacity was also observed with BEVs recovered during the later post‐recovery phase (≥ 21 days post‐onset) (Figure 4D). These results from primary PBMCs are consistent with the cytokine fluctuations and distinct dcBEV profiles observed in the recovery phase.

### 3.5 Gene Expression Alterations Within Monocyte Cell Lines Following BEV Treatment: RNA Sequencing

**FIGURE 5:**
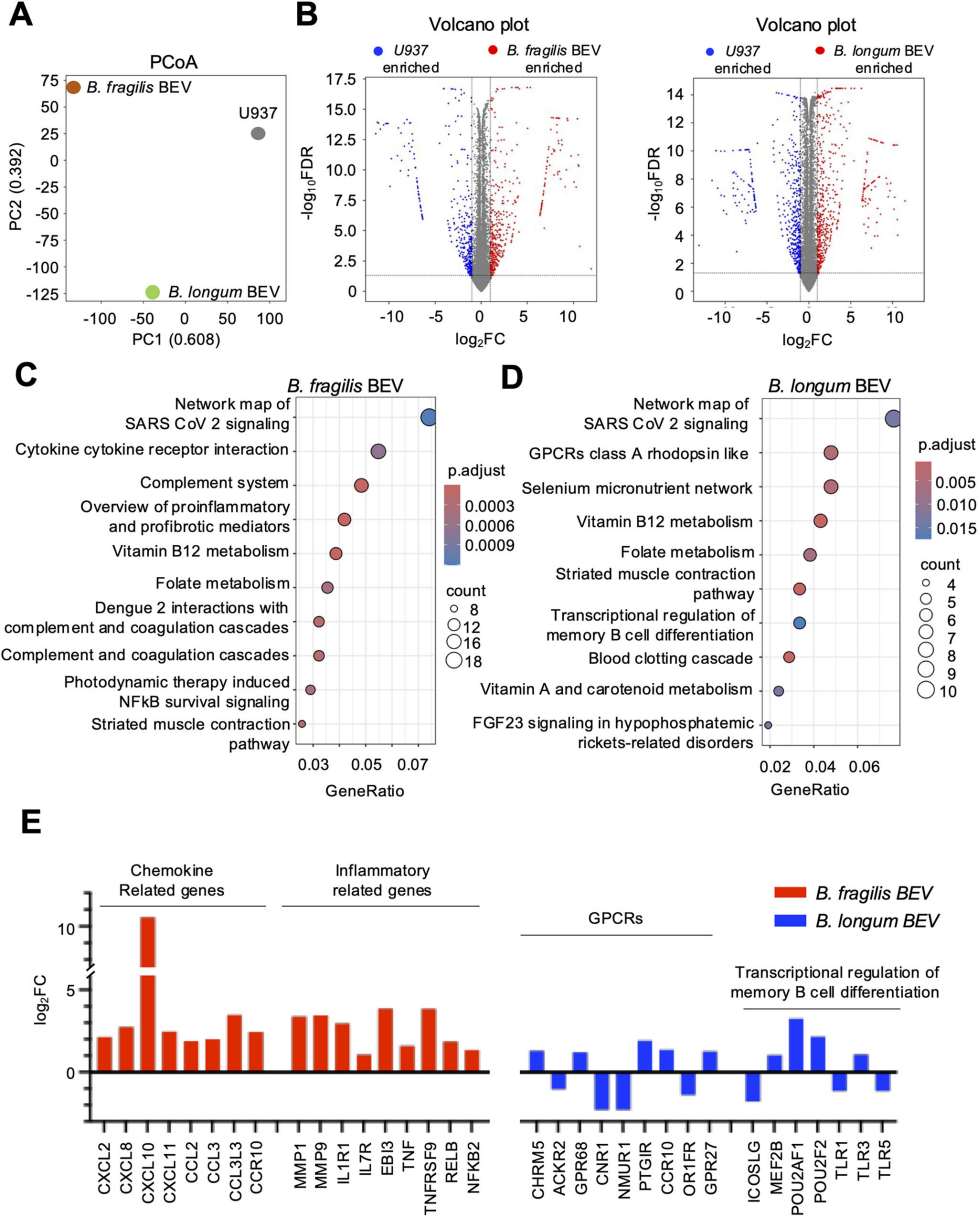
**Transcriptional profiling of U937 cells in response to specific BEV treatments**. (A) Principal coordinate analysis (PCoA) based on Bray–Curtis dissimilarity of U937 cells: untreated (black), *B. longum* BEV‐treated (green), and *B. fragilis* BEV‐treated (brown) (2,000 particles/cell). (B) Volcano plot showing differentially expressed genes (DEGs) between *B. longum*‐ and *B. fragilis* BEV‐treated cells. (C–D) Functional enrichment analysis using Gene Ontology (GO) and WikiPathways: (C) enriched pathways in *B. fragilis* BEV‐treated cells; (D) enriched pathways in *B. longum* BEV‐treated cells. (E) Representative DEGs in U937 cells across treatment groups.

Next, to investigate the effects of BEVs, whose release levels fluctuate after COVID‐19 onset, on immune cells, we examined the changes in RNA expression levels in U937 monocytes co‐cultured with BEVs released by certain strains using RNA sequencing. The bacterial strains selected for this study were *Bacteroides fragilis* and *Bifidobacterium longum*, which inhabit the human gastrointestinal tract. *B. fragilis*, a member of the Bacteroidaceae family, showed an increasing trend in BEV release after COVID‐19 onset in this study (Figure 2C and Table 1). Conversely, *B. longum* belongs to the Bifidobacteriaceae family, and BEVs derived from this family exhibited a decreasing trend in abundance during the same period (Figure 2C and Table 1). BEVs derived from *B. fragilis* (*B. fragilis*‐BEVs) and *B. longum* (*B. longum*‐BEVs) were purified from culture supernatants (Figure S4). Total RNA from U937 cells co‐cultured for 24 h with *B. fragilis*‐BEVs, *B. longum*‐BEVs, and from untreated U937 cells was analyzed. PCoA revealed distinct clustering of samples, with PCo1 and PCo2 accounting for 60.8% and 39.2% of the total variation, respectively (Figure 5A). This analysis effectively distinguished the altered RNA transcripts. Subsequently, we analyzed RNA‐level alterations in U937 cells after treatment with *B. fragilis*‐BEVs or *B. longum*‐BEVs using a volcano plot analysis. Overall, 13,937 and 13,919 genes were identified by mapping the human genome across all samples. Among these, 1,320 RNA transcripts were altered in U937 cells treated with *B. fragilis*‐BEVs (an increase in 665 genes and a decrease in 655 genes) (FDR < 0.05). Contrastingly, 969 RNA transcripts were altered in U937 cells treated with *B.longum*‐BEVs (increased, 517 genes; decreased, 452 genes) (FDR < 0.05) (Figure 5B). We conducted functional enrichment analysis using GO and WikiPathways to examine the differentially expressed RNAs. Cellular component analysis revealed that both bacteria‐derived BEV treatments induced active fluctuations in genes primarily functioning on the cell surface of U937 cells (Figure S5 and Table S6). In the functional pathway analysis, both BEV treatments demonstrated common alterations in the pathways involved, such as vitamin B12 and folate metabolism, and the blood clotting cascade (Figures 5C and 5D). Notably, in U937 cells treated with *B. fragilis*‐BEVs, the enrichment pathways were predominantly associated with some immune response pathways, including “cytokine receptor interaction” and “overview of proinflammatory and profibrotic mediators” (Figure 5C and Table S7). In U937 cells treated with *B.longum*‐BEVs, the enrichment pathways were primarily centered on “Class A or Rhodopsin‐like G protein‐coupled receptors (GPCRs)” and “Transcriptional regulation of memory B cell differentiation” (Figure 5D and Table S8). The representative DEGs in U937 cells treated with *B. fragilis*‐BEVs included chemokine‐related genes and interleukins, with notable variations in *RELB* and *NFKB2* (Figure 5E, left, and Table S7). Conversely, U937 cells treated with *B. longum*‐BEVs exhibited altered expression of multiple GPCRs, including increased expression of the extracellular proton‐sensing receptor *GPR68*, prostaglandin I2 receptor (*PTGIR*) associated with blood coagulation, and chemokine receptor *CCR10*. Additionally, changes in the expression of transcriptional coactivators *POU2AF1* and *POU2F2*, which are involved in immune responses, were observed (Figure 5E, right; Table S8). The reliability of the RNA‐sequence data was first verified by qPCR analysis, which confirmed the upregulation of *CXCL8* and *CXCL10* in U937 cells stimulated with *B. fragilis*‐derived BEVs (Figure S6).

**FIGURE 6:**
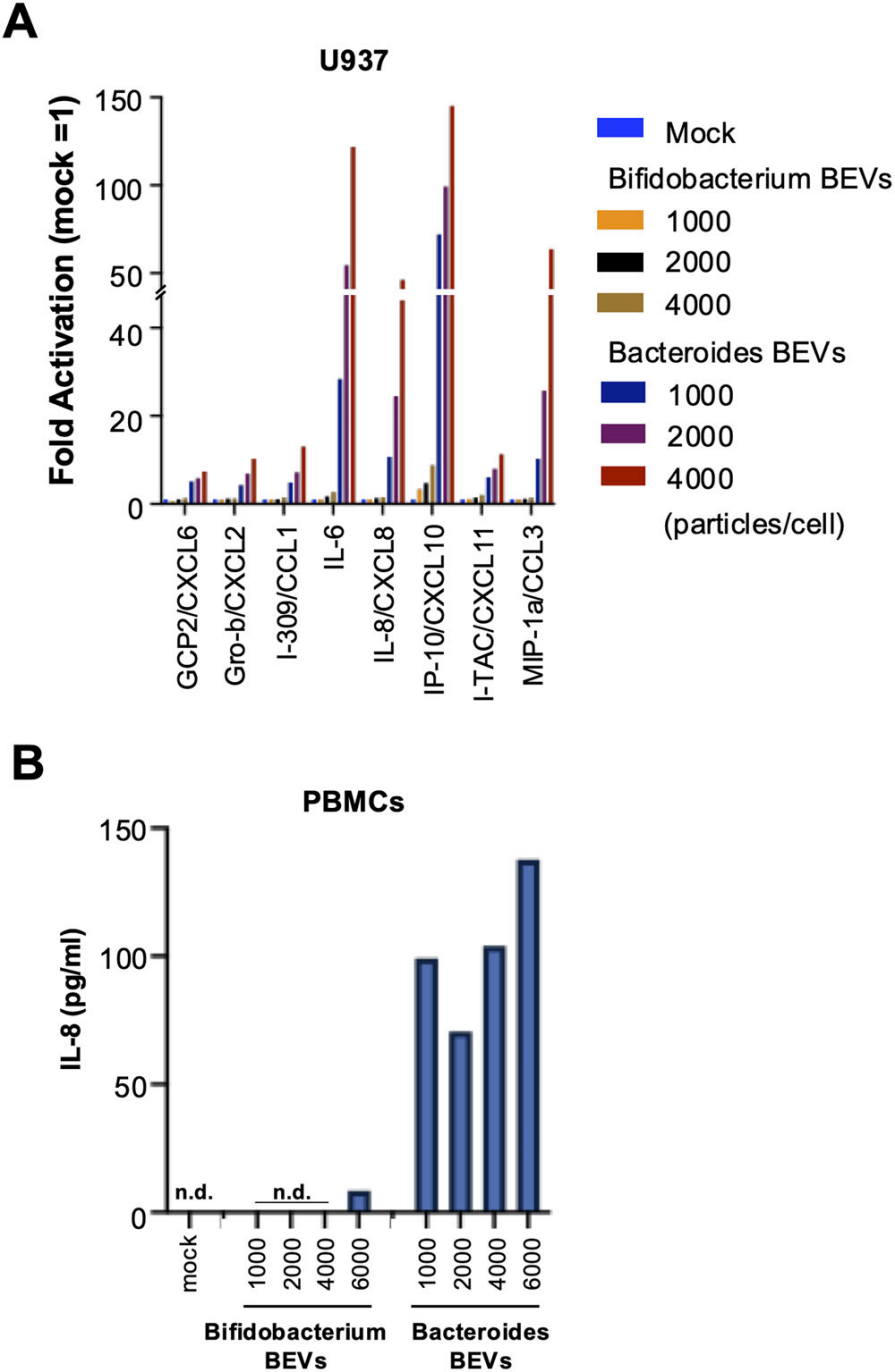
**Cytokine induction in U937 monocyte cell lines following BEV treatment**. (A) Cytokine levels in the culture supernatants of U937 cells stimulated with *Bifidobacterium longum*‐ or *Bacteroides fragilis*‐derived BEVs. (B) IL‐8 levels in supernatants of primary human PBMCs stimulated with *B. longum*‐ or *B. fragilis*‐derived BEVs. n.d., not detected.

Subsequently, we validated these changes at the protein level. Cytokine and chemokine quantification in the culture supernatant via ELISA revealed increased secretion of chemokine‐related factors (CXCL8, CXCL10, CXCL11, and CCL3), consistent with the observed RNA expression profiles (Figure 6). Furthermore, while not detected at the RNA level, *B. fragilis*‐BEVs induced the secretion of inflammatory factors including IL‐6, CXCL2, CXCL6, and CCL1 (Figure 6A). In contrast, these responses were absent following *B. longum*‐BEV treatment. This indicates that species‐specific BEVs exert distinct modulatory effects, potentially influencing host physiology through both transcriptional and translational regulation. Given that *B. fragilis*‐BEVs robustly induced chemokine expressions in the monocyte cell line, we next sought to confirm this species‐specific immunogenicity in human primary immune cells. When co‐cultured with PBMCs, *B. fragilis*‐BEVs induced a marked, concentration‐dependent upregulation of IL‐8 secretion (Figure 6B). Conversely, this prominent IL‐8 response was virtually absent following treatment with *B. longum*‐BEVs. These lines of evidence from primary PBMCs confirm that gut‐derived BEVs elicit immunogenic responses in a species‐specific manner, with *B. fragilis*‐BEVs exhibiting a distinct capacity to stimulate IL‐8 production in human immune cells.

## 4 Discussion

This study demonstrated the effects of BEVs released by gut bacteria on immune cells, using COVID‐19 as a model. The diversity of dcBEV release patterns observed during COVID‐19 progression highlights the complex nature of host–microbiome interactions during viral infection. Certain bacterial species may modulate BEV production in response to an altered gut environment, metabolic stress, and immune pressure. These diverse responses contribute to the regulation of the gut ecosystem and may influence disease progression by affecting host immune function, barrier function, and viral replication. Elucidation of the precise factors driving these distinct BEV release patterns will provide valuable insights into the role of the gut microbiota in both the pathogenesis and recovery phase of COVID‐19.

Our observation of dcBEV release patterns alongside gut microbiota alterations post‐COVID‐19 onset (Figure 2) may reflect a qualitative shift in BEV populations during dysbiosis. BEVs from patients with COVID‐19 induced alterations in cytokine secretion in the monocyte‐derived cell line U937, in contrast to BEVs from healthy individuals (Figure 3A). This suggests that changes in BEVs associated with dysbiosis may indicate alterations in their quality. One factor known to influence the release of BEVs is the SOS response. Stress conditions, such as antibiotic exposure (Pedregosa et al. 2011), oxidative stress (Takano et al. 2025), and bacteriophage infection (Quast et al. 2013), may be involved in BEV release and alteration in their content. Previous observational studies reported that viral RNA is present in stool samples from patients with COVID‐19 and often persists for an extended period (Pan et al. 2020, Zhang et al. 2020). Given physical contact between SARS‐CoV‐2 and bacteria, viral shedding in stool may influence bacterial BEVs release. Other factors that promote fluctuations in BEV content include bacterial growth stages and environmental conditions (Mao et al. 2023, Taheri et al. 2018). *Porphyromonas gingivalis* exhibits higher levels of drug‐related proteins in BEVs during the late growth phase than they do during the early growth phase (Mao et al. 2023). *Campylobacter jejuni*, the primary causative bacterium of bacterial gastroenteritis, induces changes in selective protein packaging of BEVs upon exposure to bile acids (Taheri et al. 2018). In summary, a series of changes, including viral shedding into the intestines following COVID‐19 onset, intestinal environment alterations, including metabolic secretion, and bacterial proliferation and reduction, are factors that contribute to BEV release changes. In the present analysis, the detection rate of dcBEVs was approximately 5%, a relatively low value (Figure 1F). However, it is important to acknowledge that the dye's sensitivity significantly influences fluorescence‐based single‐particle analysis, the size of encapsulated DNA/RNA fragments, and the assay's detection threshold (Takano et al. 2025). While our current detection method may underestimate nucleic acid prevalence, particularly for low‐copy or fragmented material, identifying these specific BEV subsets remains significant due to their potential roles in host‐microbe interactions. We emphasize that our findings represent the dynamics of the DNA‐containing subpopulation rather than the entire BEV population, and this limitation should be considered when interpreting our results.

In the longitudinal analysis of patients with COVID‐19, cytokine secretion sustained promotion by monocytes was observed in BEVs obtained from samples collected more than 3 weeks post‐onset (Figure 4B). These findings align with our previous report on persistent gut dysbiosis following remission from COVID‐19 (Ishizaka et al. 2024, Mizutani et al. 2022). Notably, specific samples exhibited reduced cytokine‐inducing capacity—comparable to HC—where Prevotellaceae‐derived dcBEVs predominated. While their functions remain elusive, future analyses using single‐strain cultures may identify specific pro‐ or anti‐inflammatory BEVs. Such insights will provide clues to clarify how bacterial BEVs influence disease progression and recovery. Although this study categorized BEVs based on parental origin via meta‐sequencing, investigation of the physiological roles of non‐nucleic acid‐containing BEVs remains equally essential. Additionally, it has been suggested that the microbiota may directly contribute to long COVID and serve as a potential therapeutic target (El Halabi et al. 2024, Ishizaka et al. 2024, Mendes de Almeida et al. 2023). The hypothesis that prolonged gut microbiota dysbiosis contributes to disease is plausible. If this is the case, the qualitative changes in BEVs released under these abnormal conditions, as well as the prolonged immune activation they induce, represent a potential mechanism contributing to long COVID. Although our current data do not definitively prove that BEVs drive long COVID in vivo, this hypothesis is strongly supported by our *ex vivo* validation using human primary PBMCs. Notably, while the broad‐spectrum cytokine responses captured in the U937 screening platform were more restricted in primary cells, BEVs from a subset of patients collected during both the intermediate (9–20 days) and late post‐recovery phases (≥ 21 days) retained a distinct and selective capacity to induce substantial IL‐8 secretion (Figure 4D), effectively mirroring the longitudinal dynamics observed in U937 cells (Figure 4B). This persistent, targeted pro‐inflammatory activity in primary immune cells highlights qualitatively altered BEVs as a potential functional link driving chronic immune activation during the recovery phase.

Consistent with this hypothesis, BEVs derived from single‐strain cultures of *B. fragilis* and *B. longum* induced distinct transcriptional changes in U937 cells, differentially affecting inflammatory responses and cellular signaling pathways (Figure 5 and 6A). This species‐specific immunogenicity was further substantiated in primary PBMCs, where *B. fragilis*‐BEVs elicited a marked, concentration‐dependent increase in IL‐8 production, whereas *B. longum*‐BEVs elicited no evident response (Figure 6B). Although specific GPCRs promote proliferation and differentiation of immune cells, other GPCRs may suppress immune cell activation and weaken the immune response. Changes in GPCR activity disrupt intracellular signaling pathways and are associated with inflammation, cardiovascular diseases, mental disorders, and hormonal imbalances. Loss of *B. longum* BEVs may impair expression of these receptors and potentially affect maintenance of immunological homeostasis. Furthermore, increased BEVs production by *B. fragilis* may perturb this equilibrium, leading to enhanced chemotaxis and a subsequent inflammatory response in the intestinal environment. In the BEV treatment experiment, levels of dcBEV derived from Ruminococcaceae in COVID‐19 cases correlated with the release of certain cytokines in U937 cells (Figure 3B). Members of the Ruminococcaceae family include bacteria that produce short‐chain fatty acids, which are integral to maintaining intestinal barrier homeostasis and play a significant role in sustaining intestinal health. Although short‐chain fatty acids and bacterial secondary metabolites have been shown to influence this process, it is possible that specific BEVs released from Ruminococcaceae function as regulators of intestinal homeostasis. To validate this hypothesis, it is necessary to identify BEV components, such as incorporated proteins, lipids and nucleotides, and to elucidate their mechanisms of action. While our current data demonstrate a significant correlation between specific BEV populations and cytokine induction, we acknowledge that this study is hypothesis‐generating. Future investigations using in vivo models, such as fecal microbiota transplantation or controlled administration of purified BEVs into gnotobiotic mice, will be essential to definitively establish the causal relationship between BEV composition and host immune modulation in the context of COVID‐19.

This study had some limitations. First, the cohort size of this study was relatively limited (*n* = 17 for patients and *n* = 20 for controls), which may constrain the statistical power to detect subtle microbial shifts. Consequently, no formal power calculation was performed, and the current findings should be considered exploratory. Future studies with larger sample sizes and longitudinal studies are required to validate these preliminary findings and ensure the generalizability of our results. Second, BEVs were purified using size exclusion chromatography after ultracentrifugation; however, this analysis does not completely rule out potential carryover. Although we cannot entirely rule out trace bacterial endotoxin contamination, the comparable endotoxin levels across groups—despite the differing inflammatory responses—strongly suggest that bacterial endotoxin is not the primary driver of the effects observed here (Figure S3). While the initial screening of BEV‐induced immunomodulatory effects was conducted using the U937 monocyte cell line to ensure model consistency, we extended our findings by validating key inflammatory responses in human primary cells. It is worth noting that the immunomodulatory effects in PBMCs were primarily restricted to IL‐8 secretion, with other cytokines showing less pronounced changes compared to the uniform U937 monocytic line. Since PBMCs represent a heterogeneous mixture of immune cells, this restricted response may be attributed to the diluted proportion of directly responsive cell lineages (such as monocytes) within the total peripheral blood population. Therefore, determining which distinct cell lineages—such as monocytes, T cells, or B cells—primarily drive this selective IL‐8 response remains an important next step. Furthermore, potential inter‐individual variations in donor responsiveness to BEVs must also be considered. Future studies utilizing isolated primary cell subsets or organoid models, alongside a larger cohort to account for donor‐to‐donor variability, will be valuable to comprehensively map these interactions in a more complex physiological context. Here, we focused on *B. fragilis* and *B. longum*. However, previous reports have described Toll‐like receptor‐mediated immune activation mechanisms induced by *Bacteroides thetaiotaomicron* BEVs (Gul et al. 2022), suggesting that other bacterial‐derived BEVs may also be involved in immune activation mechanisms. It is also important to note that this study classifies BEVs based solely on their DNA content.

In conclusion, this study demonstrates that alterations in BEVs associated with dysbiosis may exert immunological effects, including cytokine release by immune cells, and contribute to the pathogenesis of COVID‐19. While measuring the proportion of whole BEVs based solely on nucleic acid content has limitations, the detection of dcBEVs may serve as a useful indicator of specific pathological conditions. Although this study focused on nucleic acid‐containing BEVs, their inherent heterogeneity necessitates future research into other vesicle subpopulations to fully elucidate their roles in disease. Characterizing these diverse BEV profiles will be vital for identifying potential therapeutic targets to maintain intestinal homeostasis.

## Author Contributions

**Aya Ishizaka**: conceptualization, methodology, formal analysis, investigation, visualization, writing – review and editing. **Michiko Koga**: conceptualization, methodology, resources, writing – review and editing. **Tomoya Hayashi**: formal analysis, writing – review and editing, visualization. **Ken J Ishii**: supervision. **Hiroyuki Yamamoto**: supervision. **Hiroshi Yotsuyanagi**: supervision. **Taketoshi Mizutani**: conceptualization, methodology, formal analysis, writing – original draft, project administration, visualization, investigation.

## Funding

This research was supported by AMED under Grant Number JP25fk0410076 (A.I.), JP223fa627001 (UTOPIA Young Researcher Development Program) (A.I. and T.H.), JP223fa627001 (UTOPIA) (M.K. and H.Y.), JP223fa727001 (AMED Kunisawa G) (K.J.I.), JP223fa727002 (AMED Ishii G) (K.J.I.) and 25gm4010025h0001 (AMED FORCE) (K.J.I.). This research was also supported by JSPS KAKENHI [grant numbers JP24K11630 (A.I.), JP25K15924 (T.M.)], research grants from Taiju Life Social Welfare Foundation (A.I.), Takeda Science Foundation (A.I.), the Mochida Memorial Foundation for Medical and Pharmaceutical Research (A.I.), the Yamaguchi Education and Scholarship Foundation (A.I.), and OTC Self‐Medication Promotion Foundation (T.M.).

## Ethical Approval

This study was approved by the Ethics Committee of the Institute of Medical Sciences of the University of Tokyo (IMSUT) (approval numbers: 28‐55‐0330 and 2019‐71‐0201). All methods were conducted in compliance with relevant guidelines and regulations.

## Patient Consent Statement

The study adhered to the Declaration of Helsinki, and written informed consent for both sample collection and subsequent analysis was obtained from all participants, including patients and healthy subjects, before enrollment.

## Conflicts of Interest

All authors declare no competing interests.

## Permission to Reproduce Material From Other Sources

Not applicable.

## Clinical Trial Registration

Not applicable.

## Supporting information




**Supporting Information**: jev270341‐sup‐0001‐FigureS1.tiff

**Supporting Information**: jev270341‐sup‐0002‐FigureS2.tiff

**Supporting Information**: jev270341‐sup‐0003‐FigureS3.tiff

**Supporting Information**: jev270341‐sup‐0004‐FigureS4.tiff

**Supporting Information**: jev270341‐sup‐0005‐FigureS5.tiff

**Supporting Information**: jev270341‐sup‐0006‐FigureS6.tiff

**Supporting Information**: jev270341‐sup‐0007‐Tables.xlsx

## Data Availability

Data described in this study are openly available in the DNA Data Bank of Japan (DDBJ) (https://ddbj.nig.ac.jp/search; accession number: PRJDB12349 (16S), PRJDB35945 (BEVs data).
