# Supplementary figures and images for: Gut Microbiota‐Derived Bacterial Extracellular Vesicles in COVID‐19: Their Signature and Immunological Impact
Source: J Extracell Vesicles. 2026 Jul 23;15(7):e70341. doi: 10.1002/jev2.70341 (PMC13393066; doi:10.1002/jev2.70341)

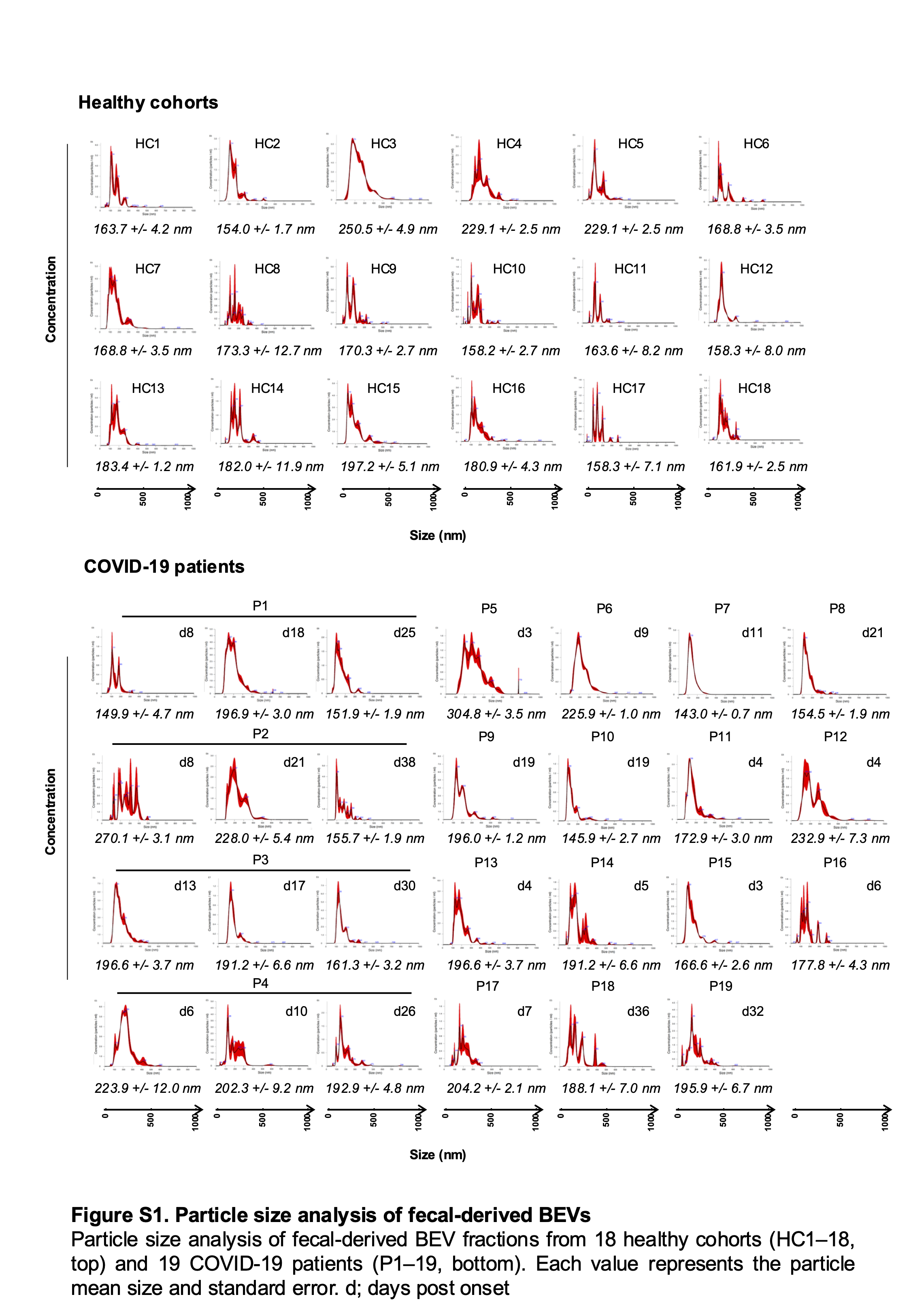

Supplement: Supplementary file 1 — Supporting Information: jev270341‐sup‐0001‐FigureS1.tiff [file JEV2-15-e70341-s005.tiff]

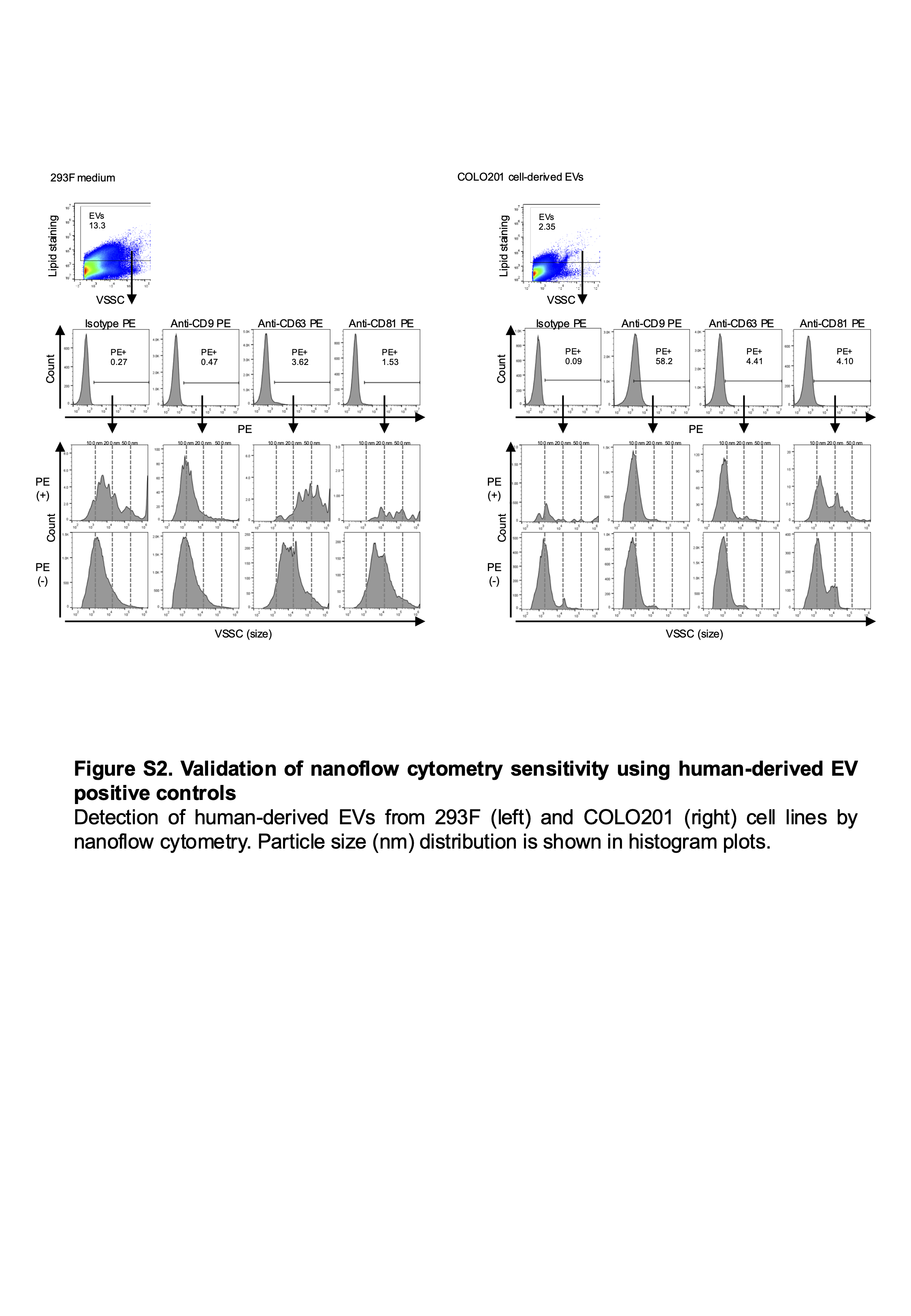

Supplement: Supplementary file 2 — Supporting Information: jev270341‐sup‐0002‐FigureS2.tiff [file JEV2-15-e70341-s002.tiff]

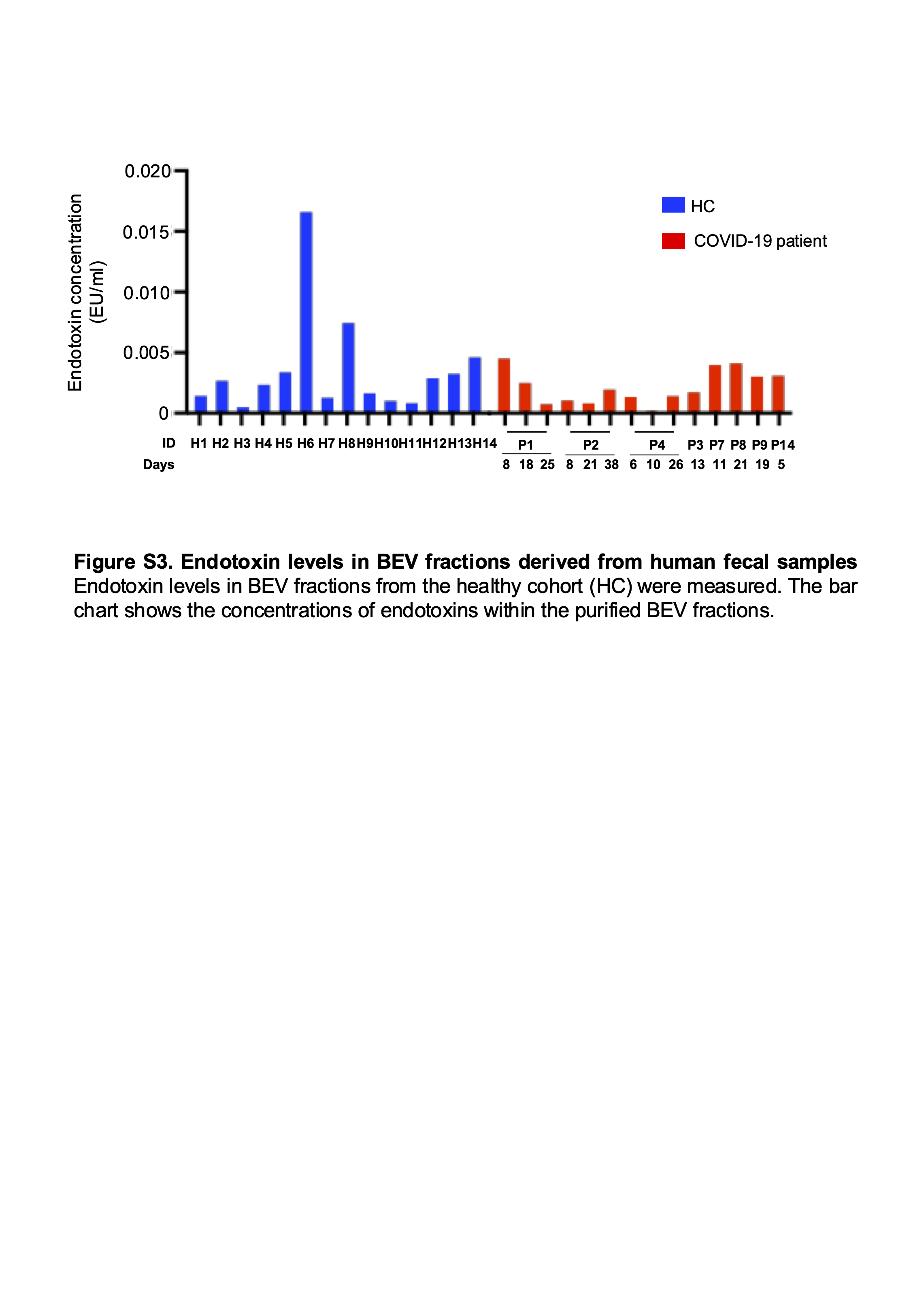

Supplement: Supplementary file 3 — Supporting Information: jev270341‐sup‐0003‐FigureS3.tiff [file JEV2-15-e70341-s003.tiff]

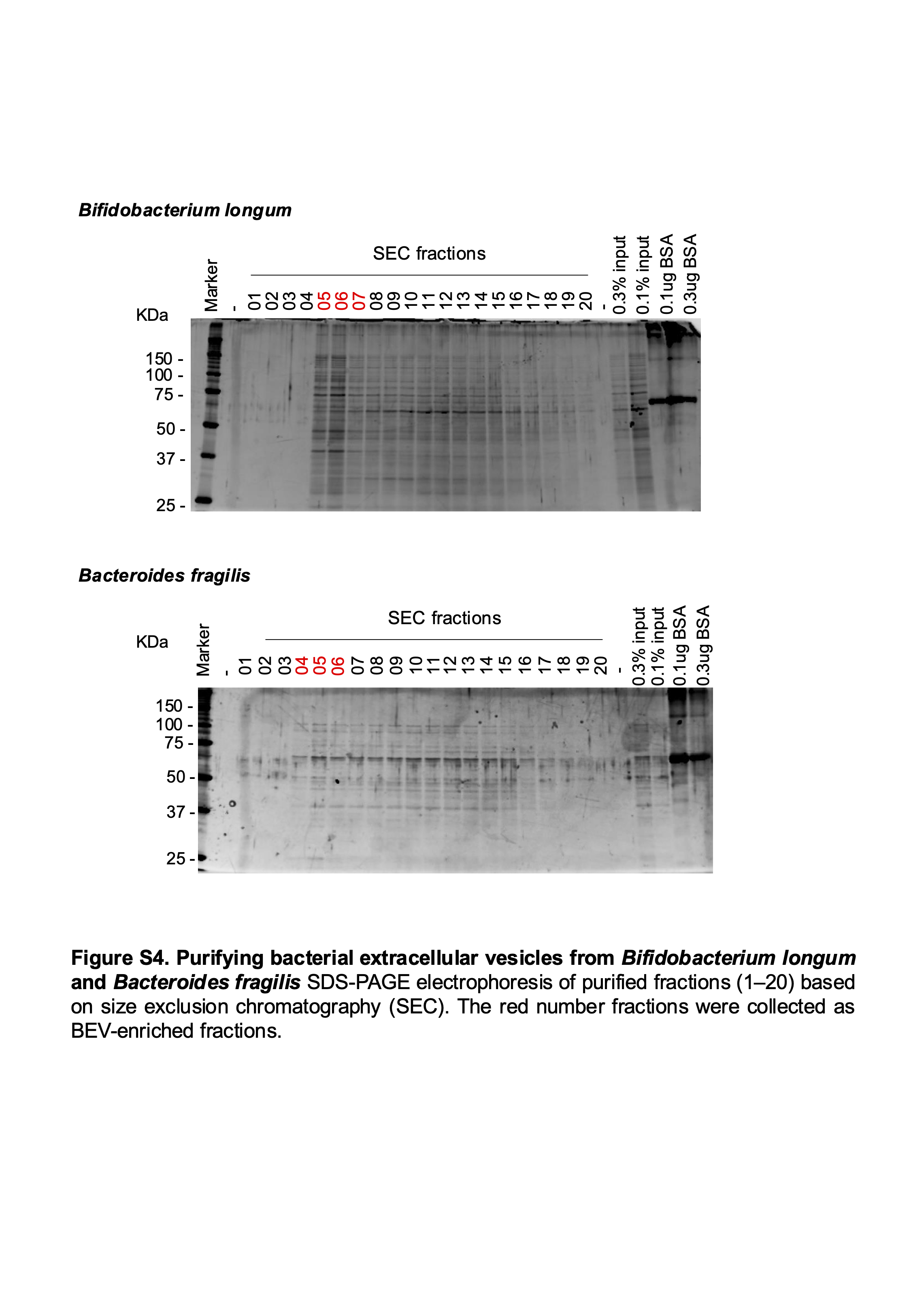

Supplement: Supplementary file 4 — Supporting Information: jev270341‐sup‐0004‐FigureS4.tiff [file JEV2-15-e70341-s001.tiff]

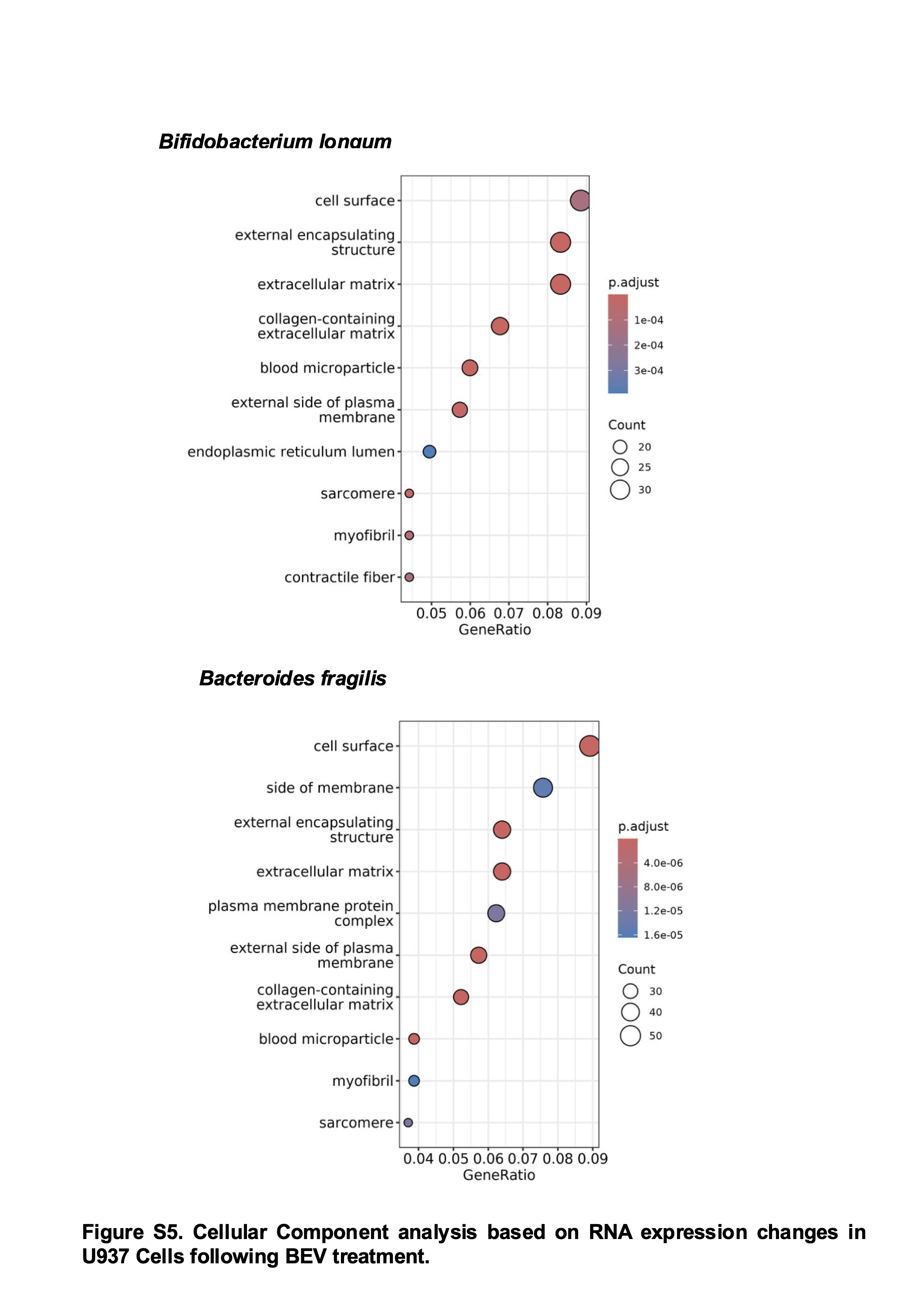

Supplement: Supplementary file 5 — Supporting Information: jev270341‐sup‐0005‐FigureS5.tiff [file JEV2-15-e70341-s004.tiff]

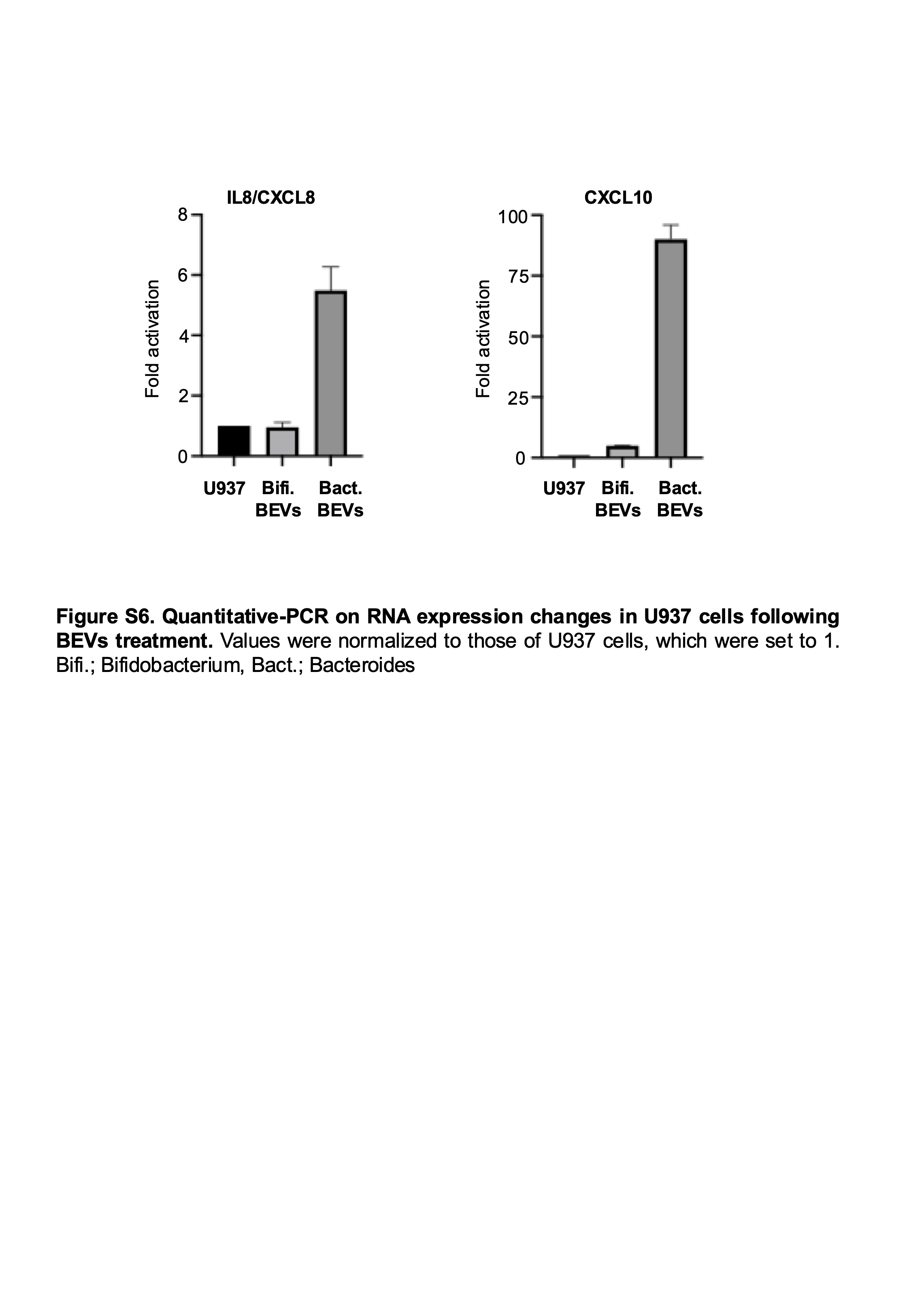

Supplement: Supplementary file 6 — Supporting Information: jev270341‐sup‐0006‐FigureS6.tiff [file JEV2-15-e70341-s007.tiff]
